# Season and outdoor temperature in relation to detection and control of hypertension in a large rural Chinese population

**DOI:** 10.1093/ije/dyu158

**Published:** 2014-08-18

**Authors:** Danting Su, Huaidong Du, Xinwei Zhang, Yijian Qian, Lingli Chen, Yaping Chen, Yu Guo, Zheng Bian, Zhengming Chen, Liming Li, Min Yu

**Affiliations:** ^1^Zhejiang Provincial Centre for Disease Control and Prevention, Hangzhou, Zhejiang, China, ^2^Clinical Trial Service Unit and Epidemiological Studies Unit (CTSU), Nuffield Department of Population Health, University of Oxford, Oxford, UK, ^3^Tongxiang Centre for Disease Control and Prevention, Zhejiang Province, China, ^4^Chinese Academy of Medical Sciences, Beijing, China and ^5^School of Public Health, Peking University Health Sciences Center, Beijing, China

**Keywords:** Blood pressure, outdoor temperature, season, hypertension detection, hypertension control, China

## Abstract

**Background:** In many Western populations, blood pressure varies moderately with season and outdoor temperature. Relatively little is known about effects of seasonal changes in blood pressure on the detection and control of hypertension in general populations, especially in low- and middle-income countries.

**Methods:** We analysed cross-sectional data of 57 375 (42% men) participants aged 30–79 (mean 52.3) years who were enrolled during 2004–08, as part of the China Kadoorie Biobank, from a rural county in the south-east costal Zhejiang Province. Analyses related daily mean outdoor temperature, obtained from local Meteorological Bureau, to mean systolic (SBP) and diastolic blood pressure (DBP), rate of newly detected hypertension and, among those with self-reported physician-diagnosed hypertension, rate of adequate blood pressure control, using multiple linear and logistic regression models.

**Results:** The overall mean blood pressure was 135.9 mmHg for SBP and 80.5 mmHg for DBP. Daily outdoor temperature ranged between −2.9 and 33.7°C, with July being the hottest month (mean 29.4°C) and January the coldest (mean 4.0°C). Comparing January (the coldest month) with July (the warmest), the differences in the adjusted SBP/DBP were 19.2/7.7 mmHg. Each 10°C lower ambient temperature was associated with 6.9/2.9 mmHg higher SBP/DBP,14.1% higher prevalence of newly detected hypertension and, among those with pre-diagnosed hypertension, 13.0% lower hypertension control rate.

**Conclusion:** In rural China, lower outdoor temperature is strongly associated with higher mean blood pressure and hypertension prevalence as well as poorer hypertension control, and should be considered when conducting population-based hypertension surveys and providing treatment for hypertensive patients.

**Key Messages**

- The significant seasonal variations in blood pressure were primarily driven by changes in outdoor temperature.
- When outdoor temperature dropped, blood pressure increase dramatically and a higher prevalence of hypertension could be detected. This should be taken into account when conducting and evaluating population-based hypertension survey.
- Lower outdoor temperature could attenuate the blood pressure control rate in people with known hypertension. This should be considered by hypertensive patients and their care/treatment providers.

## Introduction

Hypertension, or raised blood pressure, is a common, dangerous and treatable condition, but its detection and control remain a major health challenge in many parts of the world, particularly in low- and middle-income countries such as China. Several environmental and lifestyle factors are known to be associated with raised blood pressure, including cold temperature, alcohol drinking, adiposity and salt intake. In Western populations where most people have good access to adequate housing and health care, mean blood pressure in adults tends to vary only moderately with outdoor temperature.^1–4^ In China, the situation is quite different, with a recent study showing unusually large effects of outdoor temperature on blood pressure.^5^ In that study there was also a great heterogeneity in the strength of the association across different regions of China, reflecting differences not only in local climates but also in housing conditions (e.g. access to central heating) and other known determinants of blood pressure (e.g. age, adiposity, alcohol drinking).^5^ Despite these findings, questions remain about the patterns and correlates of seasonal changes of blood pressure in specific regions of China, especially in rural areas where no proper household heating during winter is available, and about their relevance for detection and control of hypertension in the general population.

To fill in this knowledge gap, we report a detailed analysis of cross-sectional data of over 50 000 adults from a rural coastal area in the south of Yangzi River, from the China Kadoorie Biobank (CKB) study.^6^ The main objectives of the study are: (i) to examine the relationships of season and outdoor temperature with blood pressure, both overall and in various subgroups of individuals; and (ii) to assess the likely changes in detection rate of hypertension and, among those with known hypertension, control rates of blood pressure by season and outdoor temperature.

## Methods

### Participants

Detailed information about the CKB study design and recruitment strategy has been reported previously.^6–8^ Participants included in the present study were recruited from Tongxiang, Zhejiang Province, which is one of the 10 study regions of CKB. All permanent residents aged 35–79 years from 104 administrative villages (accounting for 48.8% of the total Tongxiang population) were identified through local population registries and were invited to attend study assessment clinics set up specifically in each village. In total, 57 704 men and women (∼34% of those invited) participated in the CKB baseline survey which took place between August 2004 and January 2008. For the present study, 329 people were excluded because of reported use of heating in households during winter, leaving 57 375 participants in the current analysis.

The study was approved centrally by the ethics committees of the University of Oxford and the Chinese Center for Disease Control and Prevention (CDC). In addition, ethics approval was also obtained from the institutional research board at the Zhejiang Provincial CDC and Tongxiang CDC. All participants provided written informed consent.

### Data collection

Information about socio-demographic, lifestyle and medical history was collected using an interviewer-administered laptop-based electronic questionnaire.^6^ Blood pressure was measured twice by trained clinicians using a UA-779 digital monitor, after at least 5 min rest of the participant in a seated position with feet on the floor and the arm supported at heart level. The interval between the two measurements was at least 1 min. If the difference between the two measurements was more than 10 mmHg for systolic blood pressure (SBP), a third measurement was undertaken and the mean of the last two measurements was used for analyses.

In the present analysis, hypertension detection rate was defined as proportion of people who had mean SBP ≥140 mmHg or mean diastolic blood pressure (DBP) ≥90 mmHg among those without self-reported physician diagnosed hypertension. Hypertension control rate was defined as proportion of people with prior hypertension whose measured SBP <140 mmHg and DBP <90 mmHg.

Local daily outdoor temperature data collected at four time points (02:00, 08:00, 14:00 and 20:00 h) during the day for whole of the study period was provided by Tongxiang Meteorological Bureau. Mean monthly, spring (March, April and May), summer (June, July and August), autumn (September, October and November), and winter (December, January and February) outdoor temperature was calculated as the average of outdoor temperatures during the days when participants were surveyed in that month or season.

Sedentary leisure time was defined as time spent in sitting-down activities outside work, such as watching TV, reading a newspaper and using a computer. Height and weight were measured following standard protocols and BMI was calculated as weight (in kg) divided by the square of standing height (in m).^6^

### Statistical methods

Least squared means of SBP and DBP were calculated for each season (regardless of the year) and calendar month during the 3.5 years’ study period, with adjustment for age, sex and education level. In additional analyses, outdoor temperature was also adjusted for.

The least square means of SBP in each calendar month were also analysed separately in certain subgroups, including by age (in 10-year intervals), BMI categories, smoking status (for male participants only), alcohol drinking status (for male participants only), hypertensive status and fruit intake. Analyses were adjusted for mean age, sex and education level, where appropriate.

Multiple linear regression models were used to estimate changes in blood pressure per 10°C lower outdoor temperature, adjusting for age, sex and education level. In additional analyses, season, physical activity (daily hours spent on sedentary leisure activities), dietary factors (i.e. fruit and pickled vegetable intake) and BMI were adjusted for, first individually and then jointly.

Using logistic regression models, age-, sex- and education-adjusted hypertension detection and control rates were calculated for each calendar month and season (regardless of the year). To estimate changes in detection and control rates per 10°C temperature, parameters of linear regression lines were obtained from plots of age-, sex- and education- adjusted rates in each month against monthly outdoor temperature. All analyses were performed using SAS 9.3 (SAS Institute, Cary, NC, USA).

## Results

Of the participants analysed, the overall mean [standard deviation (SD)] age was 52.3 (9.9) years, 41.6% were men and 55.9% had formal education, which was higher in men than in women (73% vs 43.7%) (Table 1). Among men, 64.5% were current smokers and 38.2% reported drinking alcohol at least weekly, as opposed to 1.2% and 1.5%, respectively, in women. Overall, 35.5% participants had BMI ≥24 kg/m^2^ and 6.4%had BMI ≥28 kg/m^2^ (cut-off points often used to define overweight and obesity in China^9^), with the mean BMI slightly higher in women than in men. In both sexes, about 19% (*n* = 8216) reported having physician-diagnosed hypertension, and of these 90.7% reported taking antihypertensive drugs. A further 26.9% (29.5% in men and 25% in women) had measured SBP ≥140 and/or DBP ≥90 mmHg (i.e. newly detected hypertension) at the baseline survey. The overall mean (SD) SBP and DBP were 135.9 (21.4) and 80.5 (10.7) mmHg, respectively, with SBP and DBP both being about 2 mmHg higher in men than in women (Table 1).

**Table 1.** Main characteristics of study participants

Characteristics	Men	Women	Overall
	(*n* = 23 883)	(*n* = 33 492)	(*n* = 57 375)
Age (years), %			
30 ∼ 39	8.5	11.0	10.0
40 ∼ 49	31.0	31.8	31.4
50 ∼ 59	33.0	35.5	34.5
60 ∼ 69	20.0	16.8	18.1
70 ∼ 79	7.6	4.8	6.0
Mean (SD)	53.2 (10.2)	51.8 (9.7)	52.3 (9.9)
Highest education, %			
No normal education	27.0	56.3	44.1
Primary school	45.2	30.0	36.3
Middle school	22.2	11.4	15.9
High school or higher	5.7	2.4	3.8
Smoking, %			
Never	9.5	97.9	61.1
Ex-regular	17.7	0.3	7.6
Occasional	8.3	0.6	3.8
Current regular	64.5	1.2	27.5
Alcohol drinking, %			
Never regular	31.6	88.6	64.9
Ex-weekly	4.9	0.4	2.3
Occasional	25.3	9.6	16.1
Current weekly	38.2	1.5	16.8
BMI (kg/m^2^), %			
<18.5	5.7	6.3	6.1
18.5–23.9	61.6	56.2	58.4
24.0–27.9	27.6	30.2	29.1
≥28.0	5.1	7.3	6.4
Mean (SD)	22.7 (3.0)	23.1 (3.3)	23.0 (3.2)
Hypertension status, %			
Normotensive	51.3	55.8	54.0
Physician-diagnosed hypertension	19.2	19.1	19.1
Newly detected hypertension^a^	29.5	25.0	26.9
Antihypertensive treatment, % of diagnosed hypertension	88.8	92.1	90.7
Fruit intake, %			
≥4 days/wk	15.2	18.9	17.3
1–3 days/wk	38.6	38.1	38.3
<1 day/wk	46.2	43.0	44.4
Pickled vegetable intake, %			
≥4 days/wk	9.7	10.7	10.3
1–3 days/wk	38.8	38.5	38.6
<1 day/wk	51.5	50.7	51.0
Total physical activity, MET-h/day	33.7 (15.4)	32.4 (15.0)	32.9 (15.2)
Sedentary leisure time(h/wk), mean (SD)	13.9 (8.3)	11.4 (8.2)	12.4 (8.3)
SBP (mmHg), mean (SD)	137.0 (20.6)	135.2 (21.9)	135.9 (21.4)
DBP (mmHg), mean (SD)	81.8 (11.0)	79.6 (10.4)	80.5 (10.7)

BMI, body mass index; SBP, systolic blood pressure; DBP, diastolic blood pressure; MET, metabolic equivalent.

^a^Definition of hypertension: SBP ≥140 mmHg and/or DBP ≥90 mmHg.

Participants recruited in different seasons were very similar in terms of age and BMI. For both men and women, those recruited in spring had the highest prevalence of physician-diagnosed hypertension and antihypertensive treatment, and those in autumn had the lowest (eTable 1, available as Supplementary data at *IJE* online).

**Figure 1.**
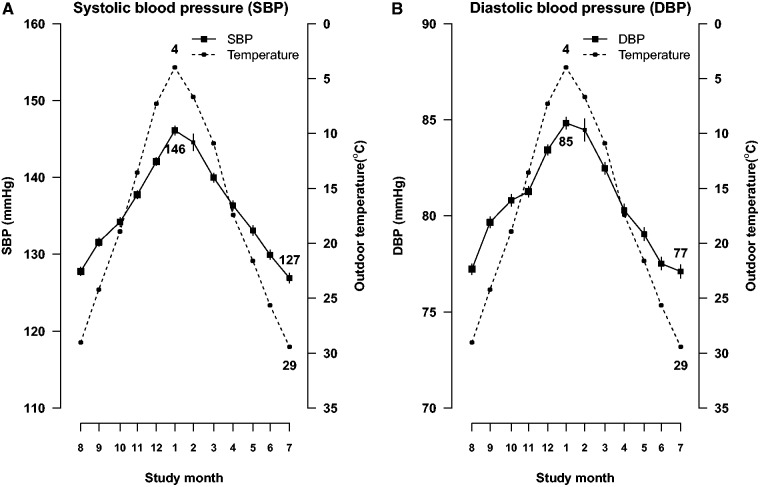
Mean (A) SBP, (B) DBP and outdoor temperature by study month. SBP, systolic blood pressure; DBP, diastolic blood pressure; CI, confidence interval. SBP and DBP values were adjusted for age, sex and education and plotted, along with mean monthly outdoor temperature, by the mean dates of survey in that month. Mean values and 95% CIs of blood pressure are shown in solid squares and vertical lines, with sizes of squares inversely proportional to the standard errors of the point estimates. Mean monthly values of outdoor temperature are shown in solid dots.

**Figure 2.**
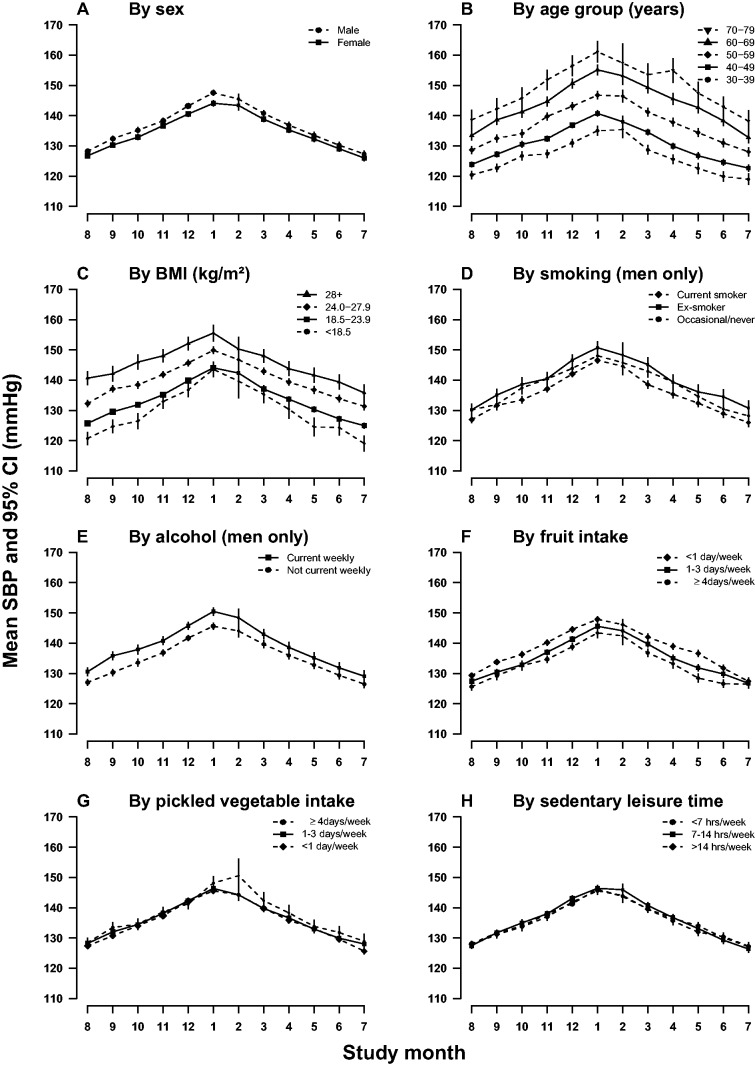
Mean SBP by study month in subgroups: (A) by sex; (B) age group (years); (C) by BMI (kg/m^2^); (D) by smoking (men only); (E) by alcohol drinking (men only); (F) by fruit intake; (G) by pickled vegetable intake; (H) by sedentary leisure time (h/day). SBP, systolic blood pressure; DBP, diastolic blood pressure; CI, confidence interval. SBP and DBP values were adjusted for age, sex and education and plotted by the mean dates of survey in that month. Mean values and 95% CIs of SBP are shown in black points and vertical lines. Sizes of points are inversely proportional to the standard errors of the point estimates.

Average daily outdoor temperature during the study period ranged from −2.9 (°C) to 33.7°C (data not shown), with the highest mean monthly temperature observed in July (29.4°C) and the lowest in January (4.0°C) (Figure 1). When outdoor temperature dropped, both SBP and DBP rose and the pattern was consistent across different years and in each month during survey period (eFigure 1, available as Supplementary data at *IJE* online). In general, SBP reached a peak (146.1 mmHg) in January and dropped to the lowest level (126.9 mmHg) in July. This variation was slightly larger in men than in women (20.3 vs 18.1 mmHg), increased with age (from 16.1 mmHg in those aged 30–39 years to 22.9 mmHg in those aged 70–79 years), and was more extreme among those with BMI <18.5 kg/m^2^ (24.5 vs 18.5–19.8 mmHg in the other three groups), but not much affected by other factors (Figure 2). The seasonal variation of blood pressure disappeared after additional adjustment for outdoor temperature (data not shown).

Table 2 shows the measured SBP, DBP, hypertension detection rate in the overall population and hypertension control rate among those with self-reported physician-diagnosed hypertension, by season. The overall winter-summer difference was 15.7 mmHg for SBP and 6.8 mmHg for DBP. Likewise, the hypertension detection rate was more than twice as high in winter (50.6%) as in summer (19.4%), whereas the converse was true for hypertension control rate, with the rate being about four times as high in summer as in winter (41.6% vs 11.0%), although the proportion of participants taking antihypertensive treatment did not vary much by season (eTable 1, available as Supplementary data at *IJE* online).

**Table 2.** Mean outdoor temperature, blood pressure and detection and control rates of hypertension by season

Season^a^	*N*	Temperature (°C)	SBP (mmHg)^b^	DBP (mmHg)^b^	Detection rate (%)^b^^,^^c^^,^^d^	Control rate (%)^b^^,^^c^^,^^e^
**Men**						
Spring	4878	16.3	137.2	82.0	36.8	27.7
Summer	5504	28.2	128.5	77.5	19.8	46.4
Autumn	6917	18.8	135.3	81.5	32.5	26.9
Winter	6584	6.3	145.1	85.6	54.3	11.9
ΔWinter-summer	–	−21.9	16.6	7.9	34.5	−34.5
**Women**						
Spring	8050	16.2	135.7	79.2	33.1	21.8
Summer	8488	28.1	127.2	76.4	18.9	38.5
Autumn	10 471	18.8	133.3	79.3	28.7	22.4
Winter	6483	5.6	142.1	82.1	47.6	10.4
ΔWinter-summer	–	−22.5	14.9	5.7	28.7	−28.1
**Overall**						
Spring	12 928	16.2	136.7	80.7	34.5	24.2
Summer	13 992	28.1	128.1	77.2	19.4	41.6
Autumn	17 388	18.8	134.5	80.6	30.3	24.2
Winter	13 067	6.0	143.8	84.0	50.6	11.0
ΔWinter-summer	–	−22.1	15.7	6.8	31.2	−30.6

SBP, systolic blood pressure; DBP, diastolic blood pressure; Δwinter-summer: winter-summer difference.

^a^Spring: March, April and May; summer: June, July and August; autumn: September, October and November; winter: December, January and February.

^b^Values were adjusted for age, sex and education, where appropriate.

^c^Hypertension was defined as SBP ≥140 mmHg and/or DBP ≥90 mmHg.

^d^Detection rate was calculated as the proportion of newly detected hypertension among those participants without prior physician-diagnosed hypertension.

^e^Control rate was calculated as the proportion of participants with measured SBP/DBP <140/90 mmHg among those with self-reported physician-diagnosed hypertension.

There was an almost perfect linear inverse association between mean monthly outdoor temperature and mean monthly BP, regardless of study year (eFigure 2, available as Supplementary data at *IJE* online). Using individual data in the model, each 10°C lower temperature was associated with 6.9/2.9 mmHg higher SBP/DBP. These associations hardly changed after additional adjustment for season, physical activity, fruit intake, pickled vegetable intake and BMI (data not shown).

**Figure 3.**
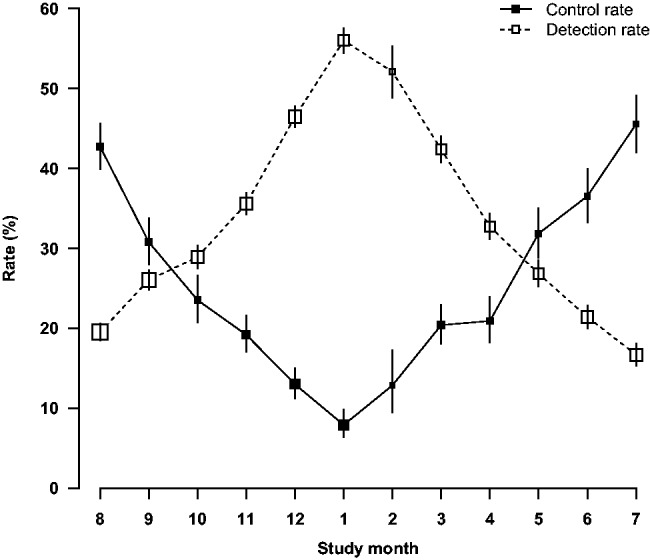
Detection and control rates of hypertension by study month. Rates were adjusted for age, sex and education, and plotted by the mean dates of survey in that month. The detection/control rates and 95% CIs are shown in open/solid squares and vertical lines. Sizes of squares are inversely proportional to the standard errors of the point estimates.

**Figure 4.**
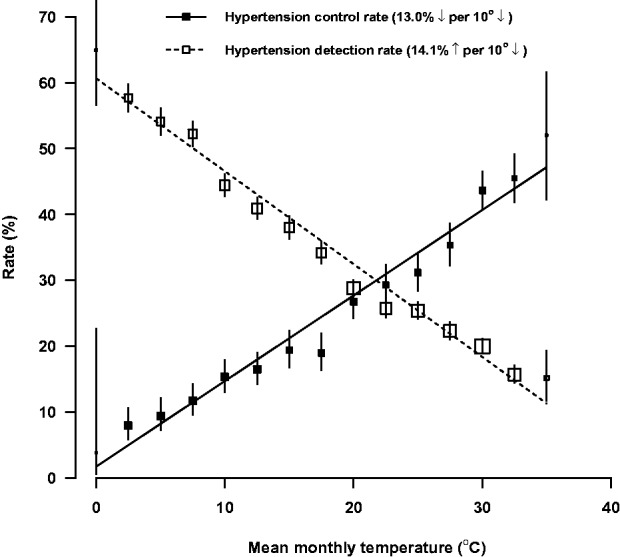
Detection and control rates of hypertension in relation to mean monthly outdoor temperature. Rates were adjusted for age, sex and education. The detection/control rates and 95% CIs are shown in open/solid squares and vertical lines. Sizes of squares are inversely proportional to the standard errors of the point estimates.

**Figure 5.**
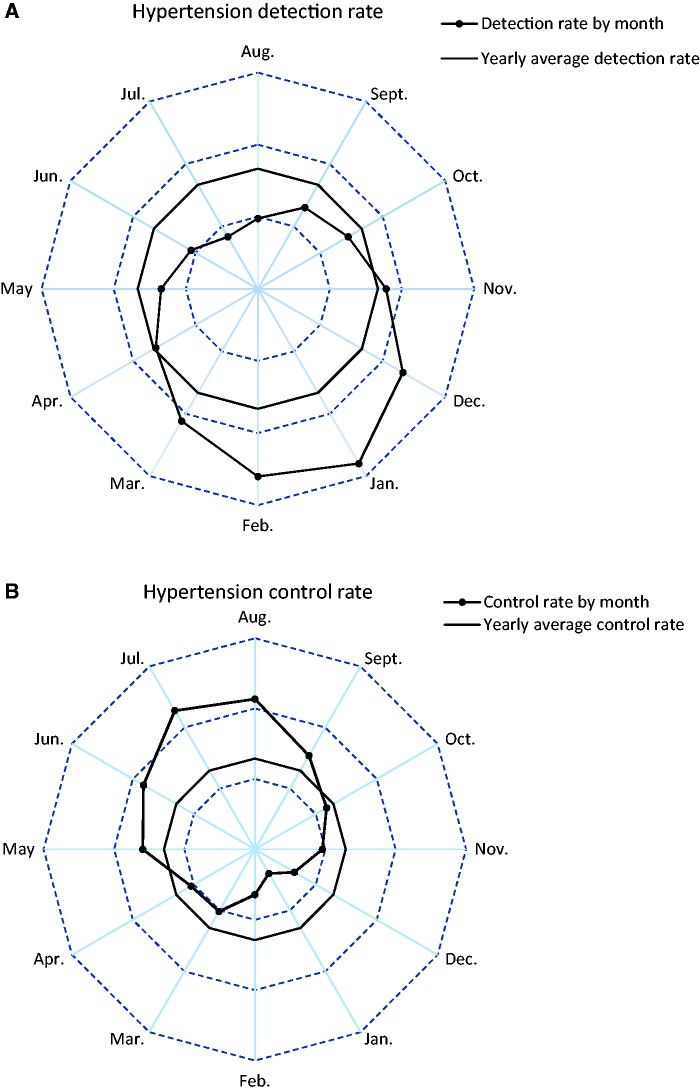
Monthly and yearly averages of hypertension detection and control rates. Rates were adjusted for age, sex and education. Solid lines with dots represent mean hypertension detection/control rates in every month; solid lines without dots represent yearly average hypertension detection/control rates; dashed lines from centre to periphery represent 20%, 40% and 60%, respectively.

The highest hypertension detection rate was seen in January (56.0%) and the lowest in July (16.7%) (Figure 3). By contrast, among people with prior history of hypertension, only about 8% had their blood pressure adequately controlled (i.e. SBP/DBP <140/90 mmHg) in January, more than 5-fold lower than that in July (45.5%). It was estimated that each 10°C lower temperature was associated with 14.1% higher hypertension detection rate but 13.0% lower hypertension control rate (Figure 4). As shown in the radar plots (Figure 5), the hypertension detection rates in months from May to October were lower, and in months from December to March were higher, than the yearly average. Similarly, the proportion of newly detected severe hypertension (i.e. SBP ≥160 mmHg and/or DBP ≥100 mmHg) was more than four times as high in cold months (December to March) as in warm months (May to October) (12.8% vs 3.1%). With regard to the monthly hypertension control rate, this was higher during May to September and lower during November to April as compared with the yearly average control rate.

## Discussion

In this large study of rural farmers from the south-east coastal region of China, we observed large seasonal variations in blood pressure, driven primarily by changes in outdoor temperature. Each 10°C lower outdoor temperature was associated with 6.9/2.9 mmHg higher SBP/DBP, a 14.1% absolute increase in hypertension detection rate and, among those with self-reported physician-diagnosed hypertension, a 13.0% absolute decrease in hypertension control rate. Comparing with the hottest month (July), participants recruited in the coldest month (January) were three times more likely to be diagnosed with hypertension (56.0% vs 16.7%), and six times less likely to have appropriate control of blood pressure if they had had hypertension (7.9% vs 45.5%).

Several studies have previously reported on the seasonal variation of blood pressure, using either spot clinic blood pressure,^10^^,^^11^ home blood pressure^12^ or mean 24-h blood pressure measurements.^3^ Most of these studies were based on Western populations where people tended to have adequate access to central heating both at home and at work during winter. So, the magnitude of the seasonal variability of blood pressure observed was much smaller than that seen in the present study. For example, in a study of 18 770 individuals living in Oslo, Norway, where the summer-winter temperature difference was about 17°C (14.8 vs −1.9°C), a 10°C lower outdoor temperature was associated only with 1.5 mmHg higher mean SBP in men and 2.4 mmHg in women.^11^ In a recent small study of 79 subjects conducted in north-east Japan where the mean monthly outdoor temperature varies between −0.7°C in January and 23.9°C in August, each 10°C lower outdoor temperature was associated with 4.0/2.8 mmHg higher SBP/DBP.^12^ In the present study the temperature variation (25.4°C between July and January and 22.1°C between summer and winter) was not much larger than that observed in the above-mentioned two studies. However, none of our study participants had access to proper household heating during winter, which may explain largely the much stronger associations observed.^13^ Other characteristics of our participants, such as a relatively low mean BMI and frequent outdoor activities, may also contribute to the observed association, but only marginally.

A few mostly hospital-based studies have also reported on the association of season or ambient temperature with detection and control of hypertension. In a small study of 275 women aged 18–40 years in Delhi, India, where the mean outdoor temperature differed by about 19°C between winter and summer, mean SBP increased by 11 mmHg from summer (114 mmHg) to winter (125 mmHg) and at the same time the detection rate of hypertension almost doubled, from 16% to 31%.^14^ In another study of 1202 male workers aged 44 ± 10 years in Tokai, Japan, where the winter-summer difference in mean outdoor temperature was ∼17°C, the overall prevalence of hypertension (SBP/DBP ≥130/85 mmHg) increased from 37% in summer to 49% in winter.^15^ A study using a database of >660 000 hypertensive adults in Southern California, USA, found that the hottest month had a 2% higher hypertension control rate than the coldest month, consistently over 5–6 years, although the temperature difference between the two was about 9°C.^16^ In another large study of 582 881 US hypertensive patients followed for 10 years, the average hypertension control rate was 6.8% higher in summer than in winter.^17^ Most of the above-mentioned studies did not assess directly the relationship of ambient temperature with hypertension detection and control rates. In a study of 73 873 patients who had visited cardiac clinics in North India over a 5-year period,^18^ there was a ∼1% decrease in the detection of new hypertension for each 10°C higher mean monthly outdoor temperature.^18^ Though consistent with overall study findings in previous studies, the strength of the association in our study was much stronger than that reported previously, implying that lower outdoor temperature will not only lead to increased blood pressure in the general population but will also attenuate the blood pressure control rate in people with known hypertension, thereby contributing to the surge of cardiovascular mortality during winter.^19^^,^^20^

Seasonal variation in blood pressure is not a new phenomenon and is probably mediated mainly by ambient temperature.^21^ Indeed in our study, seasonal variations in blood pressure disappeared completely after additional adjustment for temperature, although the limitations of the effect decomposition approach used should be recognised.^22^^,^^23^ Cold environment may affect blood pressure through several mechanisms, among which activation of the sympathetic nervous system accompanied by secretion of catecholamine and other substances involved in heat production play a major role.^13^ Several other factors, including dietary factors (e.g. fruit, alcohol and salt intake), physical inactivity and adiposity, may also potentially contribute to the seasonal variation in blood pressure. In the present study, however, there were only modest seasonal variations in measured BMI and self-reported ‘usual’ level of physical activity and intake of certain specific foodstuffs such as fruits and pickled vegetables. Consequently, additional adjustment for these variables did not materially modify the association between blood pressure and outdoor temperature (data not shown), in spite of the possibility of residual confounding.

In this study, we used the average of two blood pressure measurements made at the study clinics during daytime, which may not provide a good estimate of an individual’s usual level of blood pressure in a given condition, including season. Moreover, we used daily temperatures measured at the nearest meteorological offices from survey sites, which may not be a perfect measure of the ambient temperature to which the study participants had recently been exposed. Furthermore, no information was available on indoor temperature at home, workplace or survey clinics, nor about the time each participant spent indoors, so their effects could not be assessed directly. If, however, the measured outdoor temperatures were only partially correlated with the usual temperature to which participants were exposed during the day, our study findings may greatly underestimate the real association between ambient temperature and blood pressure.

Our study findings have implications for assessing the population burden and evaluating the clinical management of hypertension, even though the study population is not intended to be representative of the general population in China or in Zhejiang Province.^24–26^ In the past few decades, several large surveys have been undertaken in China to assess the long-term trend and burden of hypertension.^27–30^ These surveys usually lasted only a few months and tended to be done under different ambient temperature in different provinces,^29^^,^^30^ making the comparison of the results and trends difficult, if not impossible. Moreover, for practical reasons, hardly any such surveys have covered winter months, so the results from them may have substantially underestimated the real burden of hypertension in the population. In future, sampling distribution of surveys across four seasons should be considered.^31^ Similarly, for people who are already on treatment for hypertension, blood pressure should be closely monitored throughout the year, especially during winter; if necessary higher doses or additional drug(s) may be considered when temperatures drop, to achieve adequate control of blood pressure as at other times of the year. This, along with better home heating,^5^ should help to reduce the adverse effects of cold temperature on blood pressure. The continuation of long-term follow-up for mortality and morbidity in our study should help to assess any seasonal variations of cardiovascular diseases and relevance for blood pressure.

## Supplementary data

Supplementary data are available at *IJE* online.

## Funding

The China Kadoorie Biobank was supported by the: Kadoorie Charitable Foundation; Wellcome Trust; UK Medical Research Council; British Heart Foundation and Cancer Research UK. The baseline survey and first re-survey in China were supported by a research grant from the Kadoorie Charitable Foundation in Hong Kong. Follow-up of the project during 2009–14 is being supported by the Wellcome Trust in the UK (grant 088158/Z/09/Z). The project is also supported by core funding to the Clinical Trial Service Unit and Epidemiological Studies Unit (CTSU) at Oxford University from the UK Medical Research Council; the British Heart Foundation and Cancer Research UK.

## Supplementary Material

Supplementary Data

## References

[1] Rose G Seasonal variation in blood pressure in man. Nature 1961;189:235. 13743262 10.1038/189235a0

[2] Brennan PJ Greenberg G Miall WE Thompson SG Seasonal variation in arterial blood pressure. Br Med J (Clin Res Ed) 1982;285:919–23. 10.1136/bmj.285.6346.919 PMC1499985 6811068

[3] Sega R Cesana G Bombelli M Seasonal variations in home and ambulatory blood pressure in the PAMELA population. Pressione Arteriose Monitorate E Loro Associazioni. J Hypertens 1998;16:1585–92. 9856358 10.1097/00004872-199816110-00004

[4] Alperovitch A Lacombe JM Hanon O Relationship between blood pressure and outdoor temperature in a large sample of elderly individuals: the Three-City study. Arch Int Med 2009;169:75–80. 19139327 10.1001/archinternmed.2008.512

[5] Lewington S Li L Sherliker P Seasonal variation in blood pressure and its relationship with outdoor temperature in 10 diverse regions of China: the China Kadoorie Biobank. J Hypertens 2012;30:1383–91. 22688260 10.1097/HJH.0b013e32835465b5 PMC4344838

[6] Chen Z Chen J Collins R China Kadoorie Biobank of 0.5 million people: survey methods, baseline characteristics and long-term follow-up. Int J Epidemiol 2011;40:1652–66. 22158673 10.1093/ije/dyr120 PMC3235021

[7] Chen Z Lee L Chen J Cohort profile: the Kadoorie Study of Chronic Disease in China (KSCDC). Int J Epidemiol 2005;34:1243–49. 16131516 10.1093/ije/dyi174

[8] Li LM Lv J Guo Y [The China Kadoorie Biobank: related methodology and baseline characteristics of the participants]. Zhonghua liu xing bing xue za zhi 2012;33:249–55. 22613372

[9] Zhou B-F Predictive values of body mass index and waist circumference for risk factors of certain related diseases in Chinese adults: study on optimal cut-off points of body mass index and waist circumference in Chinese adults. Asia Pac J Clin Nutr 2002;1(Suppl 8):S685–93. 12046553

[10] Modesti PA Season, temperature and blood pressure: A complex interaction. Eur J Int Med 2013;24**:** 604–07. 10.1016/j.ejim.2013.08.002 23972926

[11] Madsen C Nafstad P Associations between environmental exposure and blood pressure among participants in the Oslo Health Study (HUBRO). Eur J Epidemiol 2006;21:485–91. 16858621 10.1007/s10654-006-9025-x

[12] Hozawa A Kuriyama S Shimazu T Ohmori-Matsuda K Tsuji I Seasonal variation in home blood pressure measurements and relation to outside temperature in Japan. Clin Exp Hypertens 2011;33:153–58. 21271816 10.3109/10641963.2010.531841

[13] Cuspidi C Ochoa JE Parati G Seasonal variations in blood pressure: a complex phenomenon. J Hypertens 2012;30:1315–20. 22706390 10.1097/HJH.0b013e328355d7f9

[14] Sinha P Taneja DK Singh NP Saha R Seasonal variation in prevalence of hypertension: Implications for interpretation. Ind J Public Health 2010;547–10. 10.4103/0019-557X.70537 20859042

[15] Kamezaki F Sonoda S Tomotsune Y Yunaka H Otsuji Y Seasonal variation in metabolic syndrome prevalence. Hypertens Res 2010;33:568–72. 20300109 10.1038/hr.2010.32

[16] Handler J Seasonal variability of blood pressure in California. J Clin Hypertens 2011;13:856–60. 10.1111/j.1751-7176.2011.00537.x PMC8816493 22051432

[17] Fletcher RD Amdur RL Kolodner R Blood pressure control among US veterans: a large multiyear analysis of blood pressure data from the Veterans Administration health data repository. Circulation 2012;125:2462–68. 22515976 10.1161/CIRCULATIONAHA.111.029983

[18] Narang R Wasir HS Seasonal variation in the incidence of hypertension and coronary artery disease. Int J Cardiol 1996;56:90–92. 8891811 10.1016/0167-5273(96)02708-8

[19] Sheth T Nair C Muller J Yusuf S Increased winter mortality from acute myocardial infarction and stroke: the effect of age. J Am Coll Cardiol 1999;33:1916–19. 10362193 10.1016/s0735-1097(99)00137-0

[20] Gerber Y Jacobsen SJ Killian JM Weston SA Roger VL Seasonality and daily weather conditions in relation to myocardial infarction and sudden cardiac death in Olmsted County, Minnesota, 1979 to 2002. J Am Coll Cardiol 2006;48:287–92. 16843177 10.1016/j.jacc.2006.02.065

[21] Kent ST Howard G Crosson WL Prineas RJ McClure LA The association of remotely-sensed outdoor temperature with blood pressure levels in REGARDS: a cross-sectional study of a large, national cohort of African-American and white participants. Environ Health 2011;10:7. 21247466 10.1186/1476-069X-10-7 PMC3032648

[22] Kaufman JS Maclehose RF Kaufman S A further critique of the analytic strategy of adjusting for covariates to identify biologic mediation. EP+I 2004;1:4. 15507130 10.1186/1742-5573-1-4 PMC526390

[23] Cole SR Hernan MA Fallibility in estimating direct effects. Int J Epidemiol 2002;31:163–65. 11914314 10.1093/ije/31.1.163

[24] Collins R What makes UK Biobank special? Lancet 2012;379:1173–74. 22463865 10.1016/S0140-6736(12)60404-8

[25] Manolio TA Collins R Enhancing the feasibility of large cohort studies. JAMA 2010;304:2290–91. 21098774 10.1001/jama.2010.1686 PMC3075846

[26] Rothman KJ Gallacher JE Hatch EE Why representativeness should be avoided. Int J Epidemiol 2013;42:1012–14. 24062287 10.1093/ije/dys223 PMC3888189

[27] Gu D Reynolds K Wu X Prevalence, awareness, treatment, and control of hypertension in China. Hypertension 2002;40:920–27. 12468580 10.1161/01.hyp.0000040263.94619.d5

[28] Wu Y Huxley R Li L Prevalence, awareness, treatment, and control of hypertension in China: data from the China National Nutrition and Health Survey 2002. Circulation 2008;118:2679–86. 19106390 10.1161/CIRCULATIONAHA.108.788166

[29] Zhao WH Ning G Content and method of 2010 China chronic disease surveillance. Zhonghua yu fang yi xue za zhi 2012;46**:** 692–96.23157861

[30] Wu Z Yao C Zhao D Cardiovascular disease risk factor levels and their relations to CVD rates in China – results of Sino-MONICA project. Eur J Cardiovasc Prev Rehabil 2004;11:275–83. 15292760 10.1097/01.hjr.0000136566.89429.63

[31] Psaltopoulou T Orfanos P Naska A Lenas D Trichopoulos D Trichopoulou A Prevalence, awareness, treatment and control of hypertension in a general population sample of 26,913 adults in the Greek EPIC study. Int J Epidemiol 2004;33:1345–52. 15218014 10.1093/ije/dyh249

