# Supplementary material for: Season and outdoor temperature in relation to detection and control of hypertension in a large rural Chinese population
Source: Int J Epidemiol. 2014 Aug 18;43(6):1835–45. doi: 10.1093/ije/dyu158 (PMC4276060; doi:10.1093/ije/dyu158)
Supplement: Supplementary Data [file supp_43_6_1835__index.html]

Season and outdoor temperature in relation to detection and control of hypertension in a large rural Chinese population — Season and outdoor temperature in relation to detection and control of hypertension in a large rural Chinese population — Supplementary Data 

# Season and outdoor temperature in relation to detection and control of hypertension in a large rural Chinese population

## Supplementary Data

files

**Files in this Data Supplement:**

- Supplementary Data - docx file
- Supplementary Data - docx file
