# Supplementary material for: Season and outdoor temperature in relation to detection and control of hypertension in a large rural Chinese population
Source: Int J Epidemiol. 2014 Aug 18;43(6):1835–45. doi: 10.1093/ije/dyu158 (PMC4276060; doi:10.1093/ije/dyu158)
Supplement: Supplementary Data [file supp_dyu158_ije-2014-04-0351-File003.docx]

**eTable1. Main characteristic of study participants by season**

| **Characteristics** | | **Spring** | **Summer** | **Autumn** | **Winter** |
| --- | --- | --- | --- | --- | --- |
| **Men** | |  |  |  |  |
| No. | | 4878 | 5504 | 6917 | 6584 |
| Age, years | | 53.4 (10.2) | 53.4 (10.3) | 52.7 (10.2) | 53.4 (10.1) |
| Highest education, % of high school or higher | | 6.2 | 8.2 | 4.7 | 4.3 |
| Smoking, % of current regular smokers | | 62.1 | 61.9 | 65.7 | 67.3 |
| Alcohol drinking, % of current weekly drinkers | | 36.3 | 36.4 | 39.2 | 40.1 |
| BMI, kg/m^2^ | | 23.1 (3.0) | 22.5 (3.0) | 22.6 (3.0) | 22.7 (2.9) |
| Fruit intake, % of ≥ 4 days/wk | | 16.5 | 17.0 | 14.8 | 13.1 |
| Pickled vegetable intake, % ≥ 4 days/wk | | 6.4 | 11.2 | 12.8 | 7.9 |
| Total physical activity, MET-h/day | | 32.2 (15.3) | 33.0 (15.7) | 34.1 (15.5) | 34.7 (15.1) |
| Sedentary leisure time, h/wk | | 14.6 (8.7) | 14.6 (9.0) | 13.2 (7.7) | 13.5 (7.9) |
| SBP, mmHg | | 137.5 (20.3) | 128.8 (18.1) | 135.2 (19.4) | 145.3 (21.0) |
| DBP, mmHg | | 82.0 (10.7) | 77.6 (9.9) | 81.4 (10.7) | 85.6 (11.3) |
| Hypertension status, % | |  |  |  |  |
|  | Normotensive | 49.2 | 63.5 | 56.2 | 37.5 |
|  | Physician diagnosed hypertension | 22.1 | 20.4 | 16.8 | 18.6 |
|  | Newly detected hypertension ^a^ | 28.7 | 16.1 | 27.0 | 43.9 |
| Anti-hypertensive treatment, % of diagnosed patients | | 92.7 | 91.2 | 85.6 | 86.4 |
| **Women** | |  |  |  |  |
| No. | | 8050 | 8488 | 10,471 | 6483 |
| Age, years | | 51.5 (9.6) | 52.0 (9.7) | 51.6 (9.9) | 51.9 (9.5) |
| Highest education, % of high school or higher | | 2.6 | 3.9 | 1.6 | 1.6 |
| Smoking, % of current regular smokers | | 1.2 | 1.3 | 1.1 | 1.1 |
| Alcohol drinking, % of current weekly drinkers | | 1.5 | 1.7 | 1.5 | 1.1 |
| BMI, kg/m^2^ | | 23.5 (3.3) | 23.0 (3.3) | 22.9 (3.2) | 23.0 (3.2) |
| Fruit intake, % of ≥ 4 days/wk | | 20.3 | 21.5 | 16.8 | 16.7 |
| Pickled vegetable intake, % ≥ 4 days/wk | | 7.0 | 11.7 | 12.9 | 10.6 |
| Total physical activity, MET-h/day | | 30.9 (15.1) | 31.7 (15.6) | 32.4 (14.4) | 35.0 (14.8) |
| Sedentary leisure time, h/wk | | 12.3 (8.6) | 12.2 (9.0) | 10.4 (7.4) | 10.8 (7.2) |
| SBP, mmHg | | 136.6 (21.7) | 128.4 (20.0) | 134.4 (21.6) | 143.4 (21.9) |
| DBP, mmHg | | 79.7 (10.1) | 76.9 (9.9) | 79.8 (10.2) | 82.6 (10.9) |
| Hypertension status, % | |  |  |  |  |
| Normotensive | | 53.2 | 64.4 | 58.7 | 43.4 |
| Physician diagnosed hypertension | | 21.2 | 19.9 | 17.5 | 18.2 |
| Newly detected hypertension ^a^ | | 25.6 | 15.8 | 23.8 | 38.4 |
| Anti-hypertensive treatment, % of diagnosed patients | | 94.0 | 92.7 | 88.9 | 93.2 |

BMI: body mass index; SBP: systolic blood pressure; DBP: diastolic blood pressure ^a.^ Definition of hypertension: SBP ≥ 140mmHg and/or DBP≥90mmHg

Spring: March, April, and May; summer: June, July, and August; autumn: September, October, and November; winter: December, January, and February.
