# Supplementary material for: Season and outdoor temperature in relation to detection and control of hypertension in a large rural Chinese population
Source: Int J Epidemiol. 2014 Aug 18;43(6):1835–45. doi: 10.1093/ije/dyu158 (PMC4276060; doi:10.1093/ije/dyu158)
Supplement: Supplementary Data [file supp_dyu158_ije-2014-04-0351-File004.docx]

20%

**eFigure 1. Figure Blood pressure by study month.**

Analyses were adjusted for age, sex and education. Horizontal positions of dots are the mean dates of survey in that month.

SBP: systolic blood pressure; DBP: diastolic blood pressure; CI: confidence interval.

**eFigure 2. Figure Mean blood pressure in relation to mean monthly outdoor temperature.**

SBP: systolic blood pressure; DBP: diastolic blood pressure; CI: confidence interval.

Analyses were adjusted for age, sex and education.
